# A unique GCN5-related glucosamine N-acetyltransferase region exist in the fungal multi-domain glycoside hydrolase family 3 β-N-acetylglucosaminidase

**DOI:** 10.1038/srep18292

**Published:** 2015-12-16

**Authors:** Zhen Qin, Yibei Xiao, Xinbin Yang, Jeroen R. Mesters, Shaoqing Yang, Zhengqiang Jiang

**Affiliations:** 1 College of Food Science and Nutritional Engineering, Beijing Advanced Innovation Center for Food Nutrition and Human Health, China Agricultural University, Beijing 100083, China; 2 Institute of Biochemistry, Center for Structural and Cell Biology in Medicine, University of Lübeck, Ratzeburger Allee 160, 23538, Lübeck, Germany

## Abstract

Glycoside hydrolase (GH) family 3 β-N-acetylglucosaminidases widely exist in the filamentous fungi, which may play a key role in chitin metabolism of fungi. A multi-domain GH family 3 β-N-acetylglucosaminidase from *Rhizomucor miehei* (*Rm*Nag), exhibiting a potential N-acetyltransferase region, has been recently reported to show great potential in industrial applications. In this study, the crystal structure of *Rm*Nag was determined at 2.80 Å resolution. The three-dimensional structure of *Rm*Nag showed four distinctive domains, which belong to two distinguishable functional regions — a GH family 3 β-N-acetylglucosaminidase region (N-terminal) and a N-acetyltransferase region (C-terminal). From structural and functional analysis, the C-terminal region of *Rm*Nag was identified as a unique tandem array linking general control non-derepressible 5 (GCN5)-related N-acetyltransferase (GNAT), which displayed glucosamine N-acetyltransferase activity. Structural analysis of this glucosamine N-acetyltransferase region revealed that a unique glucosamine binding pocket is located in the pantetheine arm binding terminal region of the conserved CoA binding pocket, which is different from all known GNAT members. This is the first structural report of a glucosamine N-acetyltransferase, which provides novel structural information about substrate specificity of GNATs. The structural and functional features of this multi-domain β-N-acetylglucosaminidase could be useful in studying the catalytic mechanism of GH family 3 proteins.

The general control non-derepressible 5 (GCN5)-related N-acetyltransferases (GNATs) are widespread in all kingdoms of living organisms and perform several important cellular functions through the acetylation of small molecules and protein substrates^1^. GNATs catalyse the transfer of acetyl groups from acetyl coenzyme A (AcCoA) to primary amine acceptors including aminoglycoside antibiotics^2^,^3^, glucosamine-6-phosphate (GlcN-6P)^4^,^5^, histones^6^, peptide^7^, arylamine^8^,^9^ and spermidine^10^. They were first found as aminoglycoside N-acetyltransferases in bacteria that develop antibiotic resistance to kanamycin and gentamicin^1^. Generally, there is very limited sequence conservation among members of the GNAT superfamily, in part, reflecting their capacity to bind and modify a diverse array of substrates. Previous structural studies revealed that although different enzymes of this superfamily show only moderate pairwise sequence homology, they share a common core fold comprising a central highly curved mixed β-sheet flanked on both sides by α-helices^1^,^2^.

Glucosamine N-acetyltransferase (EC 2.3.1.3) catalyses the transfer of an acetyl group from AcCoA to a glucosamine (GlcN) acceptor generating a N-acetylglucosamine. It has firstly been identified in pigeon liver extracts^11^ and participated in aminosugar metabolism. To date, these enzymes also have been found in plants and bacteria^12^,^13^. The glycan chains of the peptidoglycan of the bacterial cell wall are composed of alternating β-1,4-glycosidically linked amino sugars N-acetylglucosamine (GlcNAc) and N-acetyl-muramic acid (MurNAc)^14^. Glucosamine N-acetyltransferases from some bacteria can recover non-N-acetylated sugars (the N-acetylation of GlcN residues at the non-reducing end of peptidoglycan fragments) prior to β-N-acetylglucosaminidase cleavage^13^. However, there is no analogous glucosamine N-acetyltransferase structure that has been reported previously. In addition, whether the structural basis for substrate-specific acetylation by the fungal glucosamine N-acetyltransferase differs from the other GNATs is not clear.

The full-length β-N-acetylglucosaminidase (*Rm*Nag) from *Rhizomucor miehei* belongs to glycoside hydrolase (GH) family 3 in the CAZy classification of carbohydrate-active enzymes^15^ and the characterisation of which has been reported in our previous study^16^. *Rm*Nag is a 858-amino-acid protein with multiple distinctive structural domains and the β-N-acetylglucosaminidase activity lies in the N-terminal region. Several structures of bacterial homologues of the glycoside hydrolase domains, involved in bacterial cell wall turnover, spore germination, and induction of β-lactamase, have been determined^17^. These structures have been beneficial to design and analyse the inhibitors of GH family 3 β-N-acetylglucosaminidases^17^. For the C-terminal region of *Rm*Nag, there is no information available at present although it may constitute an acetyltransferase region. In the present study, the crystal structure of full-length *Rm*Nag has been resolved to 2.8 Å resolution to find that a unique GCN5-related N-acetyltransferase region exists in the C-terminal region of *Rm*Nag, which displays glucosamine N-acetyltransferase activity *in vitro*. Our results not only present new information on the GCN5-related N-acetyltransferase family, but also provide a new assembly of a muti-domain β-N-acetylglucosaminidase. This is the first report on a crystal structure of a fungal GH family 3 β-N-acetylglucosaminidase, representing a significant advance in our understanding of these enzymes.

## Results and Discussion

### Overall structure

**Figure 1:**
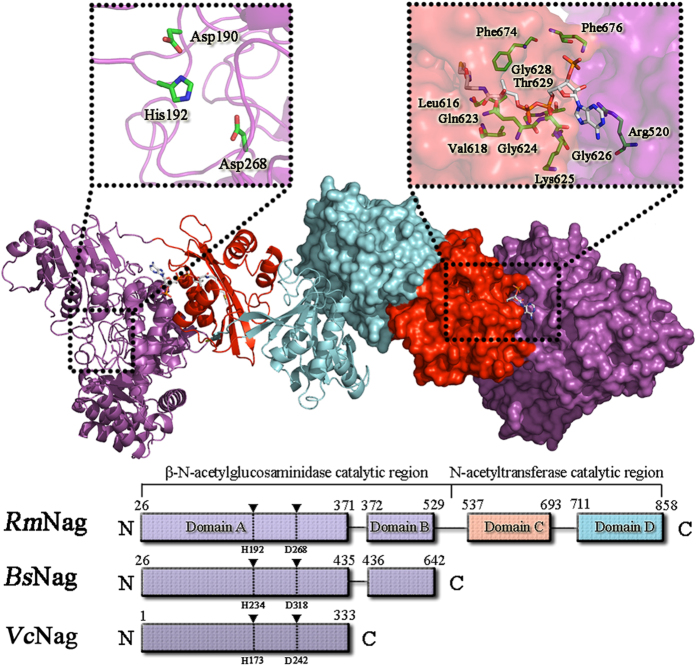
Overall structure of the full-length *Rm*Nag. One monomer of the homodimer is shown as a ribbon cartoon (upper left), while the other monomer is shown as surface representation (upper right). The β-N-acetylglucosaminidase region (NTR) is coloured in magenta; domain C and domain D of the N-acetyltransferase region (CTR) are coloured in red and cyan, respectively. AcCoA is rendered as a stick model. The arrangement of the four domains of *Rm*Nag and a comparison with the two domains of GH family 3 Nag (*Bs*Nag, PDB code: 3BMX) and single domain of GH family 3 Nag (*Vc*Nag, PDB code: 2OXN) is shown in the lower half.

The crystal structure of full-length *Rm*Nag was determined to 2.80 Å resolution. The crystallographic statistics for data collection and structure refinement are given in Table 1. The monoclinic space group of *Rm*Nag was *P*42_1_2 with two monomers in the asymmetric unit. The overall structure of *Rm*Nag is presented in Fig. 1. The *Rm*Nag monomer with approximate dimensions of 90.7 × 50.1 × 77.5 Å, consisted of a series of four separate domains (A, B, C and D). The crystal structure of full-length *Rm*Nag reveals two distinct functional regions: a β-N-acetylglucosaminidase N-terminal region (NTR, domains A and B, residues 26–529), and a N-acetyltransferase C-terminal region (CTR, domains C and D, residues 537–858) (Fig. 1). The native molecular mass of *Rm*Nag as estimated by size-exclusion chromatography (SEC) is approx. 230 kDa (data not shown), suggesting that *Rm*Nag exists as a dimer in solution. However, the β-N-acetylglucosaminidase region (NTR) and the N-acetyltransferase region (CTR) are monomeric and dimeric in solution, respectively, indicating that full-length *Rm*Nag dimerizes via the interactions owing to its CTR. Indeed, a conserved dimer interface was observed in both the crystal structures of the full-length *Rm*Nag (Fig. 1) and its CTR. Even though the buried surface area in the interface of the full-length *Rm*Nag is relatively small (900 Å^2^), it harbors 16 hydrogen bonds and 8 salt bridges. It is noteworthy that total three sulfate ions were found in the crevice of domain D of monomers, and a binding AcCoA molecule was found in the each crevice of domain C of monomers.

### β-N-Acetylglucosaminidase N-terminal region (NTR)

**Figure 2:**
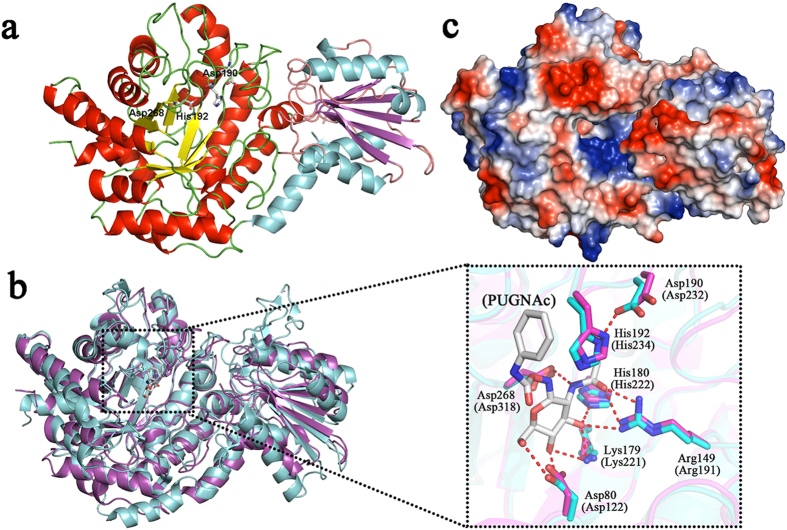
Structure of the β-N-acetylglucosaminidase region (NTR). (**a**) Cartoon representation of the β-N-acetylglucosaminidase region (NTR). The α-helices, β-strands, and loops of domain A of *Rm*Nag are coloured in red, yellow, and green, respectively and those of domain B of *Rm*Nag are coloured in cyan, magenta, and orange, respectively. The catalytic residues (His192, Asp190, and Asp268) are presented as coloured sticks. (**b**) Superimposition of *Bs*Nag-PUGNAc complex (cyan, PDB: 3NVD) and *Rm*Nag β-N-acetylglucosaminidase region (magenta, NTR). The PUGNAc is shown in stick representation. Details of the active site geometry of NTR and *Bs*Nag-PUGNAc in comparison are shown in an amplified and detailed view. Active-site residues are shown as stick models. The residues of the NTR are shown in magenta and labelled. The residues of *Bs*Nag-PUGNAc are shown in cyan and labelled in brackets. (**c**) Electrostatic-potential surface representation of the β-N-acetylglucosaminidase region (NTR) of *Rm*Nag (upper half). Red and blue surfaces represent negative and positive potentials (–10 k_B_T to + 10 k_B_T), respectively. The electrostatic surface potentials were calculated using the Adaptive Poisson-Boltzmann Solver (APBS)^47^ within the APBS plugin for PyMOL (v.1.3; Schrödinger LLC).

The β-N-acetylglucosaminidase N-terminal region (NTR) of *Rm*Nag covers two domains (A and B) (Fig. 2a). The N-terminal domain (domain A, residues 26–371) reveals a characteristic (β/α)_8_ TIM barrel, which is typical for catalytic domains of GH family 3 members (Fig. 2a). The C-terminal domain (domain B, residues 372–529) displays a 3α/6β/3α sandwich-like fold, in which a six-stranded β-sheet is sandwiched by two layers of three helices each (Fig. 2a). Even though domain B is tightly associated with domain A, domain B is located fairly far away from the catalytic site of domain A. Hence, the domain B’s function remains unclear but it is unlikely to be directly involved in the β-N-acetylglucosaminidase activity. An approximately 9.2 Å long and 5.5 Å deep catalytic pocket is located on the reverse side of the NTR molecule, allowing the binding of N-acetylglucosamine. The center of this catalytic pocket is surrounded by the residues Asp80, Arg149, Lys179, His192, Met229, Asp268 and Met297.

The catalytic nucleophile within GH family 3 has been identified as a conserved aspartate residue, while the general acid/base residue is poorly conserved. In several structures of multi-domain GH family 3 β-glucanases or β-glucosidases, the active site is composed of an aspartate nucleophile from the (β/α)_8_ TIM barrel domain, and a general acid/base catalytic glutamate residue from the 3α/6β/3α sandwich domain^18^,^19^,^20^. In contrast, in a few GH family 3 β-N-acetylglucosaminidases, the conserved aspartate nucleophile in the (β/α)_8_ TIM barrel domain is complemented by a noncanonical histidine/aspartate dyad from the same domain. This noncanonical histidine/aspartate dyad instead of a glutamate residue has been proposed to function as the catalytic acid/base, which is unique for GH family 3 members^17^,^21^. A recent study suggests that these enzymes may act preferentially as glycoside phosphorylases^22^. Histidine is used as acid-base catalyst in place of the anionic glutamate seen in other GH3 family members to provide an anionic nucleophile within the enzyme active site^22^. However, glycoside phosphorylase activity has only been detected in *Cellulomonas fimi* β-N-acetylglucosaminidase (*Cf*Nag) to date^22^. *Cf*Nag is an atypical GH family 3 β-N-acetylglucosaminidase which shows very low sequence similarity (less than 26%) with other reported GH family 3 β-N-acetylglucosaminidases. Thus, it cannot be assumed that all GH family 3 β-N-acetylglucosaminidases are glycoside phosphorylases at present.

In the structure of *Rm*Nag, a non-canonical His192/Asp190 dyad able to function as the catalytic acid/base is present on a flexible loop which is 6.3 Å apart from the conserved aspartate nucleophile (Asp268) (Fig. 2a). Furthermore, the N^δ1^ of His192 forms a hydrogen bond with O^δ1^ of Asp190 at a distance of 2.9 Å. A similar arrangement of the Asp-His-Asp triad has been reported for the β-N-acetylglucosaminidase of *Bacillus subtilis*^17^. Superposition of the β-N-acetylglucosaminidase region (NTR) onto the *Bacillus subtilis* β-N-acetylglucosaminidase in complex with PUGNAc (O-(2-acetamido-2-deoxy-D-glucopyranosylidene) amino N-phenylcarbamate; PDB accession code 3NVD) indicates a conserved overall fold and catalytic site (Fig. 2b). The overall root-mean-square deviation (RMSD) value for 513 superimposed Cα atom pairs is 1.3 Å. The importance of catalytic residues His192 and Asp268 was corroborated by site-directed mutagenesis, in which substitution of each of these residues for alanine (H192A or D268A) clearly prohibited the enzyme’s hydrolysis activity (Table 2). The structural study of *Rm*Nag provides another example that atypical subfamily β-N-acetylglucosaminidases within the GH family 3 members might use a His/Asp dyad as the catalytic acid/base.

### N-Acetyltransferase C-terminal region (CTR)

**Figure 3:**
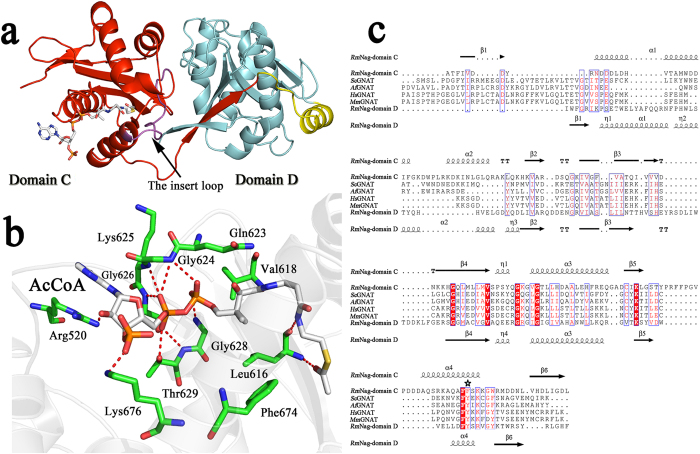
Structure of the N-acetyltransferase region (CTR). (**a**) The overall structure of the acetyltransferase region (CTR) of *Rm*Nag. Domains C and D are coloured in red and cyan, respectively. The linker α-helix is coloured in yellow. The insert loop _654_PRFFPGVPDDDAQ_666_ of domain C (absent in domain D) is highlighted in magenta. (**b**) The AcCoA ligand interacts extensively with domain C. Residues involved in the interactions are shown as coloured sticks (AcCoA carbon atoms: white; domain C carbon atoms: green; N: blue; O: red; P: orange; S: yellow) and labelled. Hydrogen bonds are represented by red dashed lines. (**c**) Sequence alignment of domains C and D with other GNAT homologues. The alignments were done using MUSCLE^45^ and the coloured figures were generated using ESPript3.0^46^. Residues coloured in red are conserved to more than 70% and residues boxed in red are completely identical. Secondary structures of domains C and D are indicated on top and bottom of the alignments, respectively. The tyrosine that is considered as a putative general acid is indicated by a star symbol. All the sequences aligned here were taken from the Genbank database (http://www.ncbi.nlm.nih.gov/genbank/): *Saccharomyces cerevisiae* GANT (*Sc*GNAT, GenBank ID: NP_116637.1), *Aspergillus fumigatus* GNAT (*Af*GNAT, GenBank ID: XP_747831.1), *Homo sapiens* GNAT (*Hs*GNAT, GenBank ID: NP_932332.1), *Mus musculus* (*Mm*GNAT, GenBank ID: NP_062298.1).

**Figure 4:**
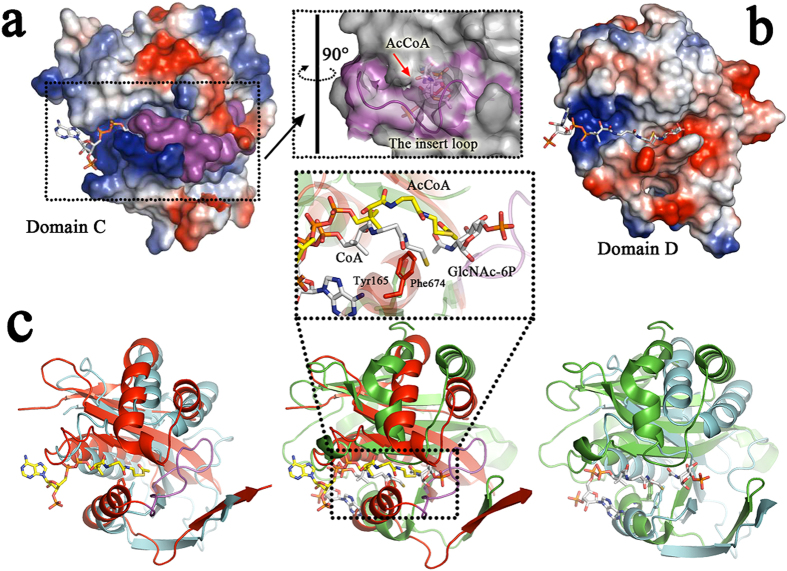
Structural comparison of the two acetyltransferase folds. (**a**) Electrostatic-potential surface of domain C of *Rm*Nag. The substrate-binding pocket of domain C is occupied by a co-purified AcCoA. Red and blue surfaces represent negative and positive potentials (–10 k_B_T to + 10 k_B_T), respectively. The electrostatic surface potentials were calculated using the APBS^47^ within the APBS plugin for PyMOL (v.1.3; Schrödinger LLC). A 90° rotation view of the substrate-binding pocket indicates that the insert loop may block the entrance of activity site of domain C. (**b**) Electrostatic-potential surface of domain D of *Rm*Nag. CoA and GlcNAc molecules are docked into the substrate-binding pocket of domain D. (**c**) Superimposition of domains C (red) and D (cyan) (left); domain C (red) and *Hs*GNA1-CoA-GlcNAc-6P complex (green; PDB: 2O28) (middle); domain D (cyan) and *Hs*GNA1-CoA-GlcNAc-6P complex (green) (right). The AcCoA, CoA and GlcNAc-6P ligands are shown as stick models (AcCoA from domain C: C: yellow, N: blue, O: red, S: brown; CoA and GlcNAc-6P from 2O28: C: white, N: blue, O: red, P: orange, S: brown). The difference of orientation between the thioester group of AcCoA from domain C and the thiol group of CoA from *Hs*GNA1-CoA-GlcNAc-6P complex (2O28) are shown in the magnified view. The insert loop of domain C is highlighted in magenta.

The N-acetyltransferase C-terminal region (CTR) of *Rm*Nag contained two structurally conserved GNAT-fold domains (C and D). A N-terminal β strand is followed by two α helices, three antiparallel β strands, and then followed by a signature central α helix, a fifth β strand, a fourth α helix and a final β strand (Fig. 3a). In spite of less than 10% sequence identity, these two domains share an overall RMSD value of 2.6 Å (for 130 superimposed Cα atom pairs) (Figs 3c and 4c). It is noteworthy that the two analogous GNAT-fold domains are arranged in a tandem array linked to the C-terminal of the β-N-acetylglucosaminidase region. Domain C and domain D are linked by one α helix. Therefore, the N-acetyltransferase region (CTR) represents a member of the subfamily of tandem GNAT structures (Fig. 3a).

A co-purified AcCoA molecule was found in the N-terminal of CTR (domain C, Ala537–Leu693). This AcCoA tightly interacts with the domain C via extensive hydrogen bonds and some hydrophobic interactions (Fig. 3b). However, this AcCoA molecule and the domain C are unlikely to be directly involved in acetyl transfer, mainly because there are no residues nearby the acetyl group that can be possibly involved in general acid/base catalysis. Several mutagenesis data have proposed a tyrosine as the proton donor for the thiolate leaving group^1^,^23^. However, sequence alignments of other GNAT homologues with the domain C indicate that the conserved tyrosine is replaced by a phenylalanine in the corresponding position of domain C (Figs 3c and 4c). In addition, this AcCoA molecule was tightly bound and its acetyl group was found to be deeply buried. It is noteworthy that a loop ^654^PRFFPGVPDDDAQ^666^ which links the fifth β strand and the fourth α helix of domain C is missing in other GNAT sequences (Fig. 3a,c). The insert loop may block the entrance of the active site of domain C and thus hinder the substrate to productively bind to the catalytic pocket (Fig. 4a).

Consequently, the acetyl transfer activity must be located within the C-terminal of CTR (domain D, Ile711–Phe858). A structural homology search at the DALI server revealed that *Rm*Nag domain D is similar in structure to *Mycobacterium tuberculosis* mycothiol synthase (PDB accession code1OZP)^24^ with an RMSD of 2.4 Å (Supplementary Table S1), despite an extremely low amino-acid sequence identity (~16%). Relevant structures are the N-acetyltransferase from *Sphaerobacter thermophilus* (3TT2), the N-acetyltransferase from *Trypanosoma brucei* (3FB3), the putative acetyltransferase from *Shigella flexneri* (2PDO), the putative N-acyltransferase form *Escherichia coli* (4QVT), the GlcN-6P N-acyltransferase from *Saccharomyces cerevisiae* (1I1D), the acyltransferase from *Listeria monocytogenes* (2OH1), and the hypothetical protein from *Drosophila melanogaster* (1SQH). The structure of GNATs is conserved from ancestral to prokaryotic and eukaryotic cells, although they show different types of substrate specificity. Superposition of the *Hs*GNA1–CoA–GlcNAc-6P complex^4^ (2O28) onto the domains C and D showed that domain D is structurally more conserved than domain C, with overall RMSD values for 130 Cα atom pairs of 1.3 Å and 2.3 Å, respectively (Fig. 4c). The substrate-binding pocket of domain D is clearly divided into two parts: the positive charged and negative charged clefts are docked with CoA and GlcNAc molecules, respectively (Fig. 4b). Most important, the conserved tyrosine (Y842 in *Rm*Nag) which has been predicted to act as the general acid to protonate the leaving thiolate anion nearly overlapped with the one of the *Hs*GNA1–CoA–GlcNAc-6P complex and it is therefore within hydrogen bond distance from the sulphur atom of a bound CoA (Fig. 4c). Mutation studies show that *Rm*Nag Y842A lost its transacetylation activity (Table 2), which confirmed the inference above.

In total, the structural analysis suggests that the AcCoA in the domain C might only possess a structural function, whereas the domain D is likely directly involved in acetyl transfer.

### N-Acetyltransferase properties of *Rm*Nag

**Figure 5:**
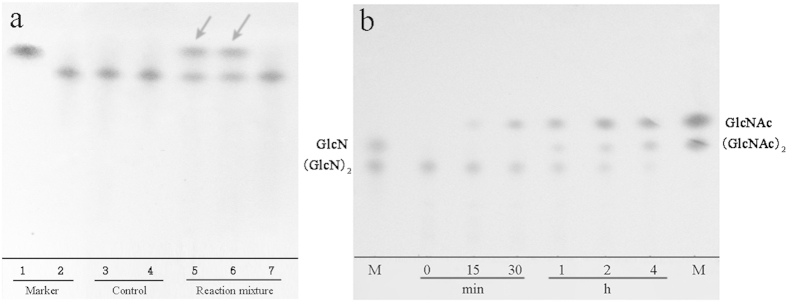
TLC analyses of the transacetylation and hydrolysis products by *Rm*Nag. (**a**) The transacetylation properties of *Rm*Nag (full length, NTR and CTR). Lane 1: marker, GlcNAc; lane 2: marker, GlcN; lane 3: control, reaction mixture without AcCoA; lane 4: control, reaction mixture without enzyme; lane 5: reaction mixture, full length *Rm*Nag; lane 6: reaction mixture, CTR; lane 7: reaction mixture, NTR. (**b**) Reaction process of chitobiose ((GlcN)_2_) hydrolysed by *Rm*Nag (full length). M: marker sugars. During the reaction progress, (GlcN)_2_ was tapered off and GlcNAc and (GlcNAc)_2_ were gradually accumulated.

The N-acetyltransferase properties of full-length *Rm*Nag, NTR and CTR toward GlcN as a substrate were investigated by thin-layer chromatography (TLC). The full-length *Rm*Nag and CTR could transfer the acetyl to GlcN using AcCoA as the co-substrate, releasing GlcNAc as final product (Fig. 5a). However, the NTR had no N-acetyltransferase activity since GlcN and AcCoA did not react in the presence of NTR. N-acetyltransferase activity of the full-length *Rm*Nag, NTR and CTR were then assayed by high performance liquid chromatography (HPLC). Full-length *Rm*Nag and CTR exhibited N-acetyltransferase activity of 115.2 and 57.7 U mg^−1^, respectively. In contrast, no N-acetyltransferase activity was detected by NTR (Table 2). On the other hand, full-length *Rm*Nag and NTR exhibited β-N-acetylglucosaminidase activity of 29.0 and 22.7 U mg^−1^, respectively. And no β-N-acetylglucosaminidase activity was detected by CTR. This result confirmed that the CTR is the N-acetyltransferase region of *Rm*Nag. Furthermore, *Rm*Nag had N-acetyltransferase activity towards both GlcN and chitosan-oligomers. It exhibited N-acetyltransferase activity range of 115.2 to 5.4 U mg^−1^ towards GlcN to (GlcN)_5_ (Table 3).

To detect the unique catalytic properties of this GH family 3 enzyme, the hydrolysis properties of full-length *Rm*Nag towards (GlcN)_2_ were further investigated (Fig. 5b). TLC analysis showed that the substrate (GlcN)_2_ declined gradually in the process of the enzymatic reaction, when AcCoA was present in the reaction mixture. Meanwhile, the products (GlcNAc)_2_ and GlcNAc increased gradually. Full-length *Rm*Nag almost completely acetylated and hydrolyzed all of the newly produced (GlcN)_2_ in 4 h to yield the end product. These results indicated that full-length *Rm*Nag could transfer the acetyl to (GlcN)_2_ using AcCoA as the co-substrate to produce (GlcNAc)_2_. The N-acetyltransferase product was then cleaved by full-length *Rm*Nag at the β-1,4 linkage in the meantime, to further yield GlcNAc monomer as the final product.

In our previous study^16^, *Rm*Nag cleaved (GlcNAc)_2_ in the absence of AcCoA. However, *Rm*Nag could not cleave (GlcN)_2_ in the absence of AcCoA (Supplementary Fig. S1). When AcCoA was present in the reaction mixture, *Rm*Nag cleaved (GlcN)_2_ to produce GlcNAc (Fig. 5b). Furthermore, both full-length and NTR displaied N-acetylgucosaminidase activity towards *p*NP-GlcNAc, but the CTR had no N-acetylgucosaminidase activity (Table 2). Thus, the transferase activity is not required for the N-acetylgucosaminidase to cleave (GlcNAc)_2_, but is processive and required for the N-acetylgucosaminidase to cleave (GlcN)_2_.

N-Acetyltransferase properties studies clearly showed that the CTR of *Rm*Nag has glucosamine N-acetyltransferase activity. It could catalyse the transfer of acetyl groups from AcCoA to primary the amine of glucosamine. These transacetylation properties were in accord with that of the glucosamine N-acetyltransferase from *Clostridium acetobutylicum*^13^. It is interesting that owing to the unique CTR, *Rm*Nag could hydrolyse chitobiose to produce acetylglucosaminide. This property is similar with the exo-β-D-glucosaminidase (EC 3.2.1.165) involved in chitosan hydrolysis^25^,^26^. Previous studies showed that some O-GlcNAc hydrolyzing enzymes (O-GlcNAcases) are also multi-domain proteins^27^,^28^, which exhibit glycoside hydrolase activity in the N-terminal domain and have a C-terminal domain with low sequence similarity to known acetyltransferases. The N-terminal region of O-GlcNAcases is a GH family 84 β-N-acetylglucosaminidase domain which specifically catalyzes the cleavage of GlcNAc from modified proteins. The C-terminal region of O-GlcNAcases is a GNAT domain which may function as a histone acetyltransferase (albeit controversial)^27^,^28^,^29^. To date, there is no information available on how the domains of these multi-domain β-N-acetylglucosaminidases interact and how the substrate is delivered from one to the other active site. The active sites of the NTR and CTR of *Rm*Nag are separated quite clearly. Also, there is no interaction of functional sites at the interfaces of the different domains derived from the current structural information of *Rm*Nag. It is thus speculated that the different domains of *Rm*Nag only possess a structural connection. The acetylated product is released into the reaction system by the N-acetyltransferase region, and then accesses the active site of the β-N-acetylglucosaminidase domain for hydrolytic cleavage. To probe this hypothesis, effects of pH and temperature on the glucosamine N-acetyltransferase activity of *Rm*Nag were examined. *Rm*Nag displayed maximal glucosamine N-acetyltransferase activity at pH 6.5 and exhibited optimal activity at 55 °C (Supplementary Fig. S2). This corresponds to the optimal pH and temperature of β-N-acetylglucosaminidase activity of *Rm*Nag, which are pH 6.5 and 50 °C, respectively^16^. These results illustrate that the β-N-acetylglucosaminidase and glucosamine N-acetyltransferase activities of *Rm*Nag are carried out at similar environmental conditions.

### Structural basis of substrate specificity of CTR

**Figure 6:**
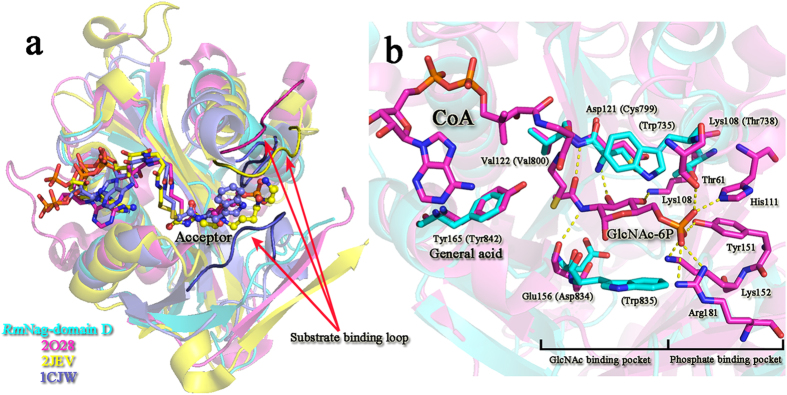
Structural comparison of *Rm*Nag N-acetyltransferase region (CTR) with different GNATs. (**a**) Superposition of *Rm*Nag-domain D with typical GNAT complexes. The Ribbon diagrams of the different proteins are colored as follows: *Rm*Nag-domain D in cyan, *Homo sapiens* glucosamine-phosphate N-acetyltransferase (PDB: 2O28) in pink, *Homo sapiens* spermidine/spermine N1-acetyltransferase (PDB: 2JEV) in yellow, *Ovis aries* serotonin N-acetyltransferase (PDB: 1CJW) in purple. The substrate binding loop of GNATs are shown in bright colors. The CoA molecules are shown in sticks and the acceptor molecules are shown in ball-and-stick models. (**b**) Superposition of CTR with the *Homo sapiens* glucosamine-phosphate N-acetyltransferase (PDB: 2O28). The CoA and the GlcNAc-6P molecules are shown in sticks. The residues involved substrate binding are shown in sticks. The residues of CTR are shown in cyan and labelled in brackets. The residues of the *Homo sapiens* glucosamine-phosphate N-acetyltransferase are shown in pink and labelled.

The GNAT superfamily is one of the largest enzyme superfamilies recognized to date and has more than 10,000 representatives from all kingdoms of life^1^. In spite of modest degrees of overall primary sequence homology, the basic structure of the GNAT fold is extraordinarily conserved, and serves two nearly universal functions: to bind the pantetheine arm of AcCoA and to polarize the carbonyl of the thioester through hydrogen bond interactions. However, diverse GNAT members have different substrate specificities, which play important roles in life activities. Therefore it is imperative to identify structural differences among various GNATs. There is no structural information available on enzymes that are functionally homologous to glucosamine N-acetyltransferases. The crystal structure of *Rm*Nag allowed us to address the molecular details of substrate binding and catalysis of the glucosamine N-acetyltransferase being different from other GNAT superfamily members. A superposition of *Rm*Nag domain D with typical GNAT complexes showed that the majority of GNATs have similar CoA binding pockets (Fig. 6a). However, the acceptor binding pockets were totally different among various GNATs. These different regions confer the substrate specificities of various GNATs. Superposition result showed that GlcN-6P N-acetyltransferase, spermidine/spermine N1-acetyltransferase and serotonin N-acetyltransferase have a loop region near the pantetheine arm terminal of CoA, which provides the residues for substrate binding (Fig. 6a). In contrast, domain D of *Rm*Nag lacked this substrate-binding loop. In addtion, an α-helix (α2) existing in this region implied that the N-acetyltransferase region of *Rm*Nag would show a unique substrate binding mode among GNATs. To reveal the structural basis of substrate specificity of CTR, the superposition of CTR with the GlcN-6P N-acetyltransferase (2O28) is shown in Fig. 6b. GlcN-6P N-acetyltransferase catalyses the transfer of acetyl groups from AcCoA and recognizes analogous acceptors compared to the glucosamine N-acetyltransferase (GlcN and GlcN-6P, respectively). The substrate binding pocket of the GlcN-6P N-acetyltransferase can be divided into two parts: a GlcNAc binding pocket (Lys108, Asp121, Val122 and Glu156) and a phosphate binding pocket (Thr61, His111, Tyr151, Lys152 and Arg181). In contrast, CTR lacks the entire phosphate-binding pocket (Fig. 6b). This structural architecture excludes CTR from binding GlcN-6P. On the other side, CTR possesses a GlcNAc binding pocket, which is similar to the GlcN-6P N-acetyltransferase. Residues Thr738, Cys799, Val800 and Asp834 of CTR superpose on the corresponding residues of the GlcN-6P N-acetyltransferase’s GlcNAc binding pocket. Furthermore, two aromatic residues (Trp735 and Trp835) are located in the GlcNAc binding pocket of CTR, which may stack against the pyranose ring, forming the hydrophobic sugar-binding platform (Fig. 6b). This architecture forms the structural basis of substrate specificity of CTR to bind glucosamine.

CTR consists of two fused GNAT domains, only one of which is functional. This cross-talk of the domains is less reported in GNATs. The other two reported tandem GNATs are *Saccharomyces cerevisiae* N-myristoyltransferase^30^ and *Mycobacterium tuberculosis* mycothiol synthase^24^. On the basis of the two previous studies and the structural information of *Rm*Nag, the function of the AcCoA ligand bound to the N-terminal domain (domain C) is still unclear, it may act as an effector molecule or function to stabilize the domain^24^,^30^. Furthermore, some tandem GNATs (2O28 and 1OZP) form unique active sites at their dimer interfaces. The similarity of the subunit interface of typical GNAT proteins and the interface between the two GNAT domains of these tandem GNATs suggests that the progenitor of tandem GNAT may have arisen from gene duplication and fusion of a homodimeric GNAT, followed by structural rewiring through mutation and selection^24^.

### Phylogenetic and sequence analyses of *Rm*Nag

Typical GH family 3 members consist of two diverse domains^31^,^32^. The GH family 3 β-N-acetylglucosaminidases have been proved to possess an Asp-His-Asp triplet catalytic core^17^,^21^. Unlike other members of the GH family 3, all of the three catalytic residues were detected in the N-domain of the enzymes ((β/α)_8_ TIM barrel domain). Some β-N-acetylglucosaminidases of the GH family 3 even completely lack a C-domain^33^. Phylogenetic analysis of GH family 3 β-N-acetylglucosaminidase (or putative β-N-acetylglucosaminidase) ies (Supplementary Fig. S3). Ssequences generated a tree in which all 41 sequences were placed into two subfamilubfamily 1 contained 21 sequences, including several β-N-acetylglucosaminidases from gram negative bacteria, such as *Salmonella typhimurium, Escherichia coli,* and *Burkholderia cenocepacia.* It is noteworthy that all three structurally characterized β-N-acetylglucosaminidases of subfamily 1 are single domain β-N-acetylglucosaminidases. All the members of subfamily 1 lack the C-terminal domain sequences which are typical of GH family 3 proteins. Subfamily 2 covered 20 sequences, including *Rm*Nag and other proteins from fungi, gram positive bacteria and microalgae. Subfamily 2 members are multi-domain proteins, which contain C-terminal domain sequences of other representative GH family 3 proteins. However, different from other typical GH family 3 proteins, the C-terminal domains of subfamily 2 β-N-acetylglucosaminidases lacked the catalytic residues and thus did not take part in the catalysis. The subfamilies can be distinguished by differences in the sequence pattern next to the conserved PVV(L)D motif in the N-terminal domain. *Rm*NAG and other β-N-acetylglucosaminidases from fungi possess an additional region that has been predicted to be a N-acetyltransferase region. These fungal proteins can be divided into an independent clade from subfamily 2. This unique glucosamine N-acetyltransferase region was found to widely exist in the C-terminal of GH family 3 β-N-acetylglucosaminidases from fungi such as *Mucor*, *Rhizopus*, *Aspergillus*, *Penicillium*, and *Parastitella* deposited at the Genebank.

### Physiological role of *Rm*Nag

From the N-acetyltransferase properties and sequence analyses of *Rm*Nag, the most possible physiological function of *Rm*Nag is an involvement in the fungal chitin metabolism and cell wall rearrangement. Chitin, composed of β-1,4-linked N–acetylglucosamine (GlcNAc) units, is present as an important component in the cell wall of fungi, which is essential for fungi to maintain cell structure integrity^34^. The acetylation level of the fungal cell wall chitin is dynamic and the chitin polymer usually contains some GlcN units^35^. In the fungal chitin metabolism, terminal GlcN units from chitin (or related oligosaccharides) are hardly cleaved by β-N-acetylglucosaminidases. Therefore, an exo-β-D-glucosaminidase (EC 3.2.1.165) activity is needed in the fungal chitin metabolism.

According to the classification of carbohydrate-active enzymes (CAZy)^15^, exo-β-D-glucosaminidases are grouped into three GH families: 2, 9, and 35. However, no fungal exo-β-D-glucosaminidase has been characterized to date. On the basis of the above consideration, multi-domain β-N-acetylglucosaminidases may play this role in fungal chitin metabolism instead of an exo-β-D-glucosaminidase. This is consistent with findings that the unique glucosamine N-acetyltransferase region widely exists in the C-terminal region of GH family 3 β-N-acetylglucosaminidases from fungi.

## Conclusions

In this study, the full-length multi-domain GH family 3 β-N-acetylglucosaminidase from *R. miehei* (*Rm*Nag) was structurally characterized to resolution of 2.80 Å. The crystal structure of *Rm*Nag displays four separate domains, which belong to two distinct functional regions. From the three-dimensional structure of *Rm*Nag, a unique tandem array linking GCN5-related N-acetyltransferase region exists in the C-terminal of *Rm*Nag. This N-acetyltransferase region was identified as a glucosamine N-acetyltransferase region by structural and functional analysis. The structural basis for its substrate binding pocket, which is feature required for the proper function of this protein, has been identified based on structural homology within the GNAT members. These results should be useful in studying the catalytic mechanism of other GH family 3 proteins and provide novel information on the GCN5-related N-acetyltransferases.

## Methods

### Cloning, gene expression and protein purification

DNA sequences encoding full-length *Rm*Nag, N-terminal region (NTR, Met1-Asn529) and C-terminal region (CTR, Val530-Phe858), were amplified by PCR using *Pfu* DNA polymerase (Invitrogen) from the genomic DNA of *R. miehei* CAU432^36^. The resulting fragments were then cloned into a modified Sumo-pET28a(+) expression vector using *Bam*HI and *Not*I sites. All genes were expressed in the *Escherichia coli* strain BL21-*Gold* (DE3). Briefly, the cultures were grown in 2 × YT medium at 37 °C until the optical density at 600 nm was up to 0.8. Expression was induced by adding isopropyl-β-D-thiogalactopyranoside (IPTG) to a final concentration of 0.5 mM and incubation at 25 °C overnight. The selenomethionyl (SeMet) derivative of CTR was prepared using the method of methionine-biosynthesis pathway inhibition. Cells were harvested by centrifugation and lysed by sonication in buffer A (50 mM Tris-HCl pH 8.0, 20 mM imidazole and 300 mM NaCl). The lysate was ultracentrifuged at 20,000 rpm for 60 min at 4 °C, and the supernatant was applied onto a pre-equilibrated 5 mL HisTrap HP column (GE Healthcare), followed by washing with 100 mL buffer A. The proteins were eluted with buffer B (50 mM Tris-HCl pH 8.0, 300 mM NaCl and 500 mM imidazole), and the eluted samples were mixed with SUMO-protease. The cleavage was performed at 4 °C overnight. The cleaved samples were further purified by size-exclusion chromatography (HiLoad 16/60 Superdex 200; GE Healthcare) equilibrated with buffer C (10 mM Tris-HCl pH 7.5, 150 mM NaCl and 5 mM DTT). The recombinant proteins contain two additional amino acids (Gly and Ser) at their N-terminals.

The site-directed mutagenesis (D268A, H192A and Y842A) were performed directly on the Sumo-pET28a(+) expression vector containing the *Rm*Nag gene, by the quick-change method with the *Fast Mutagenesis System* site-directed mutagenesis kit (TransGen Biotech, China). Primers used in gene amplification and site-directed mutagenesis are listed in Supplementary Table S2. The desired mutants were selected, sequenced and transformed into *E. coli* strain BL21-*Gold* (DE3) for expression.

### Crystallization and data collection

Crystals were screened using a Phoenix robot (Art Robbins Instruments) with the sitting-drop, vapor-diffusion method at 20 °C and drops containing 0.3 μL of protein solution plus 0.3 μL of reservoir solution. The following commercial screening kits were used: SaltRx^TM^, PEG/Ion^TM^, Index^TM^, Crystal Screen^TM^, and PEGRx^TM^ (all from Hampton Research). Crystals of both *Rm*Nag full-length and CTR appeared in a drop containing 0.1 M Bis-Tris (pH 5.5–6.5), 2 M (NH_4_)_2_SO_4_. Optimized crystals suitable for diffraction were grown in drops containing 1.5 μL of protein solution and 1.5 μL of reservoir solution (1.3–1.6 M (NH_4_)_2_SO_4_ , 0.1 M Bis-Tris pH 5.5–6.5) at 20 °C. Crystals were soaked in reservoir solution supplemented with 20% glycerol, and then vitrified in liquid nitrogen. Diffraction data of the CTR and full-length *Rm*Nag were collected at 100 K using beamline BL14.2 at BESSY (Berlin, Germany) and P11 at PETRA III (Hamburg, Germany), respectively. Indexing, integration and scaling of data were carried out with XDS^37^. The program XPREP (Bruker) was used to further analyse and prepare datasets for structure solution and refinement. Statistics of the datasets are summarized in Table 1.

### Phase determination, model building and refinement

The crystal structure of the *Rm*Nag CTR was determined by the single-wavelength anomalous diffraction (SAD) method. Phase calculations and initial model building were carried out by the program AutoSol from the PHENIX suite^38^. Thereafter, model building and refinement were performed using Coot^39^ and Refmac5^40^, respectively. To determine the *Rm*Nag full-length structure, a model representing its NTR was prepared firstly by modifying the coordinates of *Bacillus subtilis* N-acetylglucosaminidase (PDB code: 2BMX), guided by an amino acid sequence alignment. The resultant NTR model, together with the model of CTR, were employed as search models in PHASER^41^. The refinement was performed by Refmac5 with automatically determined TLS groups and NCS restraints (chain A to B) introduced. The structure was refined to 2.8 Å with *R*_work_ and *R*_free_ values of 0.229 and 0.254, respectively. The geometry of the final models was scrutinized using MolProbity^42^. Structural homologs of CTR were identified at the DALI server^43^. Secondary structure elements were identified employing DSSP^44^. The structure cartoons were prepared in PyMOL (v.1.3; Schrödinger LLC). The sequence alignments were created with MUSCLE^45^ and ESPript^46^. Coordinates and structure factors of *Rm*Nag have been deposited at the Protein Data Bank under accession number 4ZM6.

### Enzyme assay and transacetylation properties

N-acetyltransferase activity was determined by high performance liquid chromatography (HPLC) using glucosamine as the substrate (acceptor) and N-acetylglucosamine as the product. The reaction mixtures containing 20 μL of 50 mM substrate in 50 mM Bis-Tris pH 6.5, 20 μL of 50 mM AcCoA (as acetyl donor) and 20 μL of suitably diluted enzyme (full length or CTR) were incubated at 55 °C for 10 min. The reactions were then terminated by boiling for 5 min and substrate/product determined by HPLC (Agilent1200, Agilent, USA) equipped with a gel-filtration sugar-D KS-802 column (Shodex, Japan). The N-acetylglucosamine was eluted with water at a flow rate of 0.6 mL min^–1^ at 65 °C and the absorbance monitored at 210 nm. One unit of enzyme activity was defined as the amount of enzyme required to produce 1 μmol N-acetylglucosamine per minute under the above mentioned conditions.

β-N-acetylglucosaminidase activity was determined spectrophotometrically with *p*NP-GlcNAc as the substrate^16^. One unit of enzyme activity was defined as the amount of enzyme required to liberate 1 μmol of *p*NP per minute under the assay conditions.

The transacetylation properties of *Rm*Nag were investigated by analysis of reaction products from the glucosamine and chitobiose. To test the N-acetyltransferase ability of *Rm*Nag (full length, NTR and CTR), 20 μL of suitably diluted enzyme was added to 50 mM glucosamine in 50 mM Bis-Tris pH 6.5 with 20 μL of 50 mM AcCoA, and then incubated at 30 °C for 4 h. Samples withdrawn at different times were immediately boiled for 5 min, and then analysed by thin-layer chromatography (TLC). The control samples were reaction mixtures without AcCoA or without enzymes, and incubated at the same conditions. The enzymatic reaction supernatants were spotted onto TLC plates (silica gel 60 F_254_; Merck, Germany) using n-butanol: methanol: ammonia: water (5:4:2:1 v/v/v/v) as the developing solution. The TLC plates were visualized after dripping the plate in methanol containing 2% (v/v) concentrated H_2_SO_4_, following by heating at 130 °C for a few minutes. The synergy of N-acetyltransferase and β-N-acetylglucosaminidase ability of *Rm*Nag was investigated by analyzing the hydrolytic products from the chitobiose. Reaction mixtures (10 μL) containing 50 mM chitobiose and 50 mM AcCoA were incubated in 50 mM Bis-Tris pH 6.5 with suitably diluted enzyme (full length) at 30 °C for 4 h. Samples withdrawn at different times were terminated by boiling for 5 min, then analysed by TLC as above.

## Additional Information

**Accession codes**: The atomic coordinates and structure factors for the crystal structure of *Rm*Nag have been deposited at the Protein Data Bank (http://www.pdb.org) under accession code 4ZM6.

**How to cite this article**: Qin, Z. *et al.* A unique GCN5-related glucosamine N-acetyltransferase region exist in the fungal multi-domain glycoside hydrolase family 3 β-N-acetylglucosaminidase. *Sci. Rep.* **5**, 18292; doi: 10.1038/srep18292 (2015).

## Supplementary Material

Supplementary Information

## Figures and Tables

**Table 1:** Data collection and refinement statistics.

	CTR, Se-derivative	*Rm*Nag, full-length
*Data collection statistics*		
X-ray source	BL14.2, BESSY, Berlin	P11, PETRA III, Hamburg
Detector	MX-225 CCD	Pilatus 6M fast
Wavelength (Å)	0.9798	1.0331
Space group	*P*4_2_22	*P*42_1_2
Unit-cell dimensions (Å)	*a* = *b* = 82.16, *c* = 180.03	*a* = *b* = 245.03, *c* = 94.52
Resolution range (Å)	49.05–2.26 (2.38–2.26)	88.19–2.80 (2.95–2.80)
Number of unique reflections	30364 (4277)	71082 (10201)
Completeness (%)	99.8 (98.5)	99.9 (99.3)
Mean I/sigma(I)	19.1 (2.6)	29.4 (4.3)
Multiplicity	26.9 (27.0)	26.2 (27.3)
*R*_merge_(%)^a^	11.4 (140.9)	11.8 (78.6)
*R*_measure_(%)^b^	11.6 (143.6)	12.0 (80.0)
*R*_pim_(%)^b^	2.2 (27.3)	2.3 (15.1)
Wilson B-factor (Å^2^)	40.2	62.6
*Refinement statistics*		
Resolution range (Å)		86.63-2.80
No. of reflections in working/test		66830/3561
*R*_work_ (%)^c^		22.9 (28.9)
*R*_free_ (%)^c^		25.4 (31.7)
Protein atoms		13603
No. of water molecules		114
No. of ions		3
RMSD in bond lengths (Å)		0.013
RMSD in bond angles (°)		1.70
RMSD B-factor for bonded atoms		2.67
Average B-factor (Å^2^)		
Overall		51.11
Protein		50.55
Solvent		54.16
Ions		111.14
Ligands		69.84
Ramachandran plot (%)		
Most favored		94.3
Additionally allowed		5.2
Outliers		0.5
Rotamer outliers (%)		5.1
C-beta outliers (%)		0.25
Clashscore		6.76
Overall score		2.29

Values in parentheses represent the highest resolution shell. ^a^*R*_merge_* = Σ*_*hkl*_*Σ*_*i*_*|I(hkl)*_*i*_*– < I(hkl) > |/Σ*_*hkl*_*Σ*_*i*_*I(hkl)*_*i*_ where *I(hkl)*_*i*_ is the intensity of reflection *hkl* and its symmetry equivalents and* < I(hkl) > *is the average intensity over all equivalent reflections.

^b^*R*_measure_: multiplicity-weighted *R*_merge_; *R*_pim_: precision-indicating *R*_merge_.

^c^*R = Σ||F*_*o*_*| − |F*_*c*_*||/Σ|F*_*o*_*|*. *|F*_*o*_*|* and *|F*_*c*_*|* are amplitudes of the observed and calculated structure factors, respectively. *R*_work_ is the *R* value for reflections used in refinement, whereas *R*_free_ is the *R* value for 5% of the reflections which are selected randomly and are not included in the refinement.

**Table 2:** Hydrolysis and transacetylation activities of wild type and variants of *Rm*Nag.

*Rm*Nag	Specific activity (U mg^−1^)	Relative activity (%)
Wild type	Hydrolysis activity	
	29.0	100
NTR	22.7	78.3
CTR	None^a^	0
D268A	None	0
H192A	None	0
	Transacetylation activity	
Wild type	115.2	100
CTR	57.7	50.1
NTR	None	0
Y842A	None	0

^a^No activity was detected.

**Table 3:** Transacetylation activity of *Rm*Nag towards GlcN and chitosan-oligomers.

Substrate	Specific activity (U mg^−1^)^a^	Relative activity (%)
GlcN	115.2	100
(GlcN)_2_	89.3	77.5
(GlcN)_3_	13.8	12.0
(GlcN)_4_	10.7	9.3
(GlcN)_5_	5.4	4.7

^a^Specific activity towards chitosan-oligomers was determined by HPLC. Reactions were performed in 50 mM Bis-Tris buffer pH 6.5 at 55 °C for 10 min.
