# Supplementary material for: A unique GCN5-related glucosamine N-acetyltransferase region exist in the fungal multi-domain glycoside hydrolase family 3 β-N-acetylglucosaminidase
Source: Sci Rep. 2015 Dec 16;5:18292. doi: 10.1038/srep18292 (PMC4680927; doi:10.1038/srep18292)
Supplement: Supplementary Information [file srep18292-s1.doc]

**Supplementary Material**

**A unique GCN5-related glucosamine N-acetyltransferase region exist in the fungal multi-domain GH3 β-N-acetylglucosaminidase**

**Zhen Qin^1^*, Yibei Xiao^2‡^*, Xinbin Yang^1^, Jeroen R Mesters^2^, Shaoqing Yang^1^ & Zhengqiang Jiang^1†^**

^1^ College of Food Science and Nutritional Engineering, Beijing Advanced Innovation Center for Food Nutrition and Human Health (Beijing), China Agricultural University, Beijing 100083, China, and ^2^ Institute of Biochemistry, Center for Structural and Cell Biology in Medicine, University of Lübeck, Ratzeburger Allee 160, 23538, Lübeck, Germany

| PDB code | Protein | Z-score | RMSD (Å) | Identity (%) |
| --- | --- | --- | --- | --- |
| 1OZP | mycothiol synthase (Rv0819) | 14.4 | 2.4 | 16 |
| 3TT2 | GCN5-related N-Acetyltransferase | 13.7 | 2.3 | 19 |
| 3FB3 | N-acetyltransferase | 13.0 | 3.1 | 16 |
| 2PDO | putative Acetyltransferase | 13.0 | 3.0 | 21 |
| 4QVT | predicted N-acyltransferase | 12.9 | 3.0 | 23 |
| 1I1D | GlcN-6P N-acyltransferase | 12.8 | 3.1 | 12 |
| 2OH1 | Acetyltransferase | 12.7 | 2.5 | 13 |
| 1SQH | hypothetical protein | 12.7 | 2.8 | 13 |

**Table S1** Structural comparisons of *Rm*Nag-domain D with structurally similar proteins at DALI server.

| Primers | Primer sequence (5′→3′) | Base pairs (bp) |
| --- | --- | --- |
| *Rm*Nag-up^a^ | CGCGGATCCATGACGGTCGGTAACGATGACAA | 32 |
| *Rm*Nag-down | ATAAGAATGCGGCCGCTTAAAAGTGACCAAGGCGGTAACTTCT | 43 |
| NTR-down | ATAAGAATGCGGCCGCTTACACGTTGGGTATGCTGATAG | 39 |
| CTR-up | CGCGGATCCGACGATGCTATCGCGCCAGC | 29 |
| H192A-up^b^ | TGCAACTGACTCAGCCTTGGATGTGC | 26 |
| H192A-down | GCTGAGTCAGTTGCAGTGTCTCCGTGGC | 28 |
| D268A-up | TATCATTACTGCTTGTCTAGAAATG | 25 |
| D268A-down | GCAGTAATGATAACGCCTTTGTATCC | 26 |
| Y842A-up | AACTTTTAGATTTCGCCAGTCGAGTTG | 27 |
| Y842A-down | GCGAAATCTAAAAGTTCCACCCAGTC | 26 |

**Table S2** Primers used in this study. ^a^Restriction enzyme sites incorporated into the primers are underlined. ^b^Mutations are indicated in box.


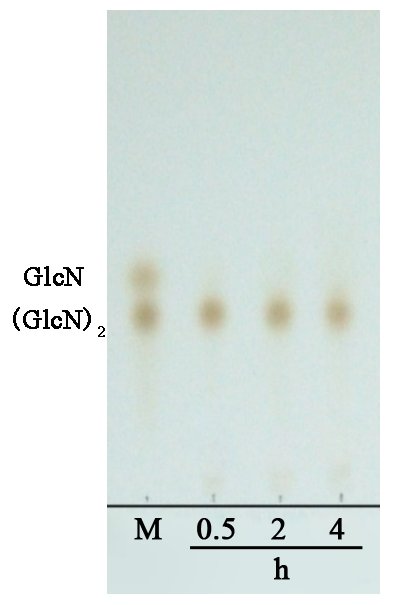


**Supplementary Figure S1**

TLC analyses of the hydrolysis product of (GlcN)_2_ by *Rm*Nag. M: marker sugars. Reaction mixtures containing 50 mM (GlcN)_2_ and suitably diluted enzyme (full length) were incubated in 50 mM Bis-Tris pH 6.5 with at 30°C for 4 h.


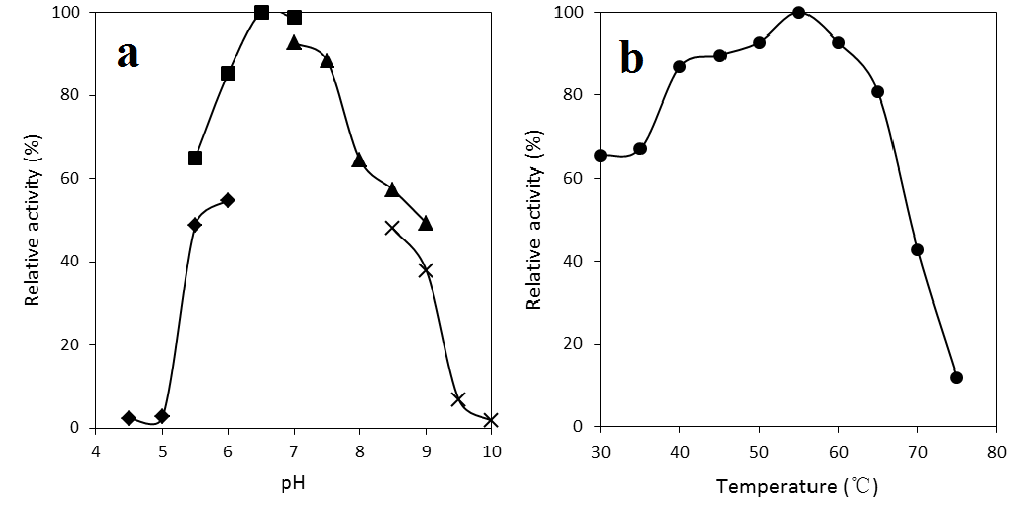
**Supplementary Figure S2**

Optimal pH (a) and optimal temperature (b) of the N-acetyltransferase activity of *Rm*Nag. Buffers were citrate buffer (◆), Bis-Tris buffer (■), Tris-HCl buffer (▲) and glycine-NaOH buffer (×). To determine the optimal temperature, the activities were measured over a temperature range of 30–75°C in 50 mM Bis-Tris buffer pH 6.5.


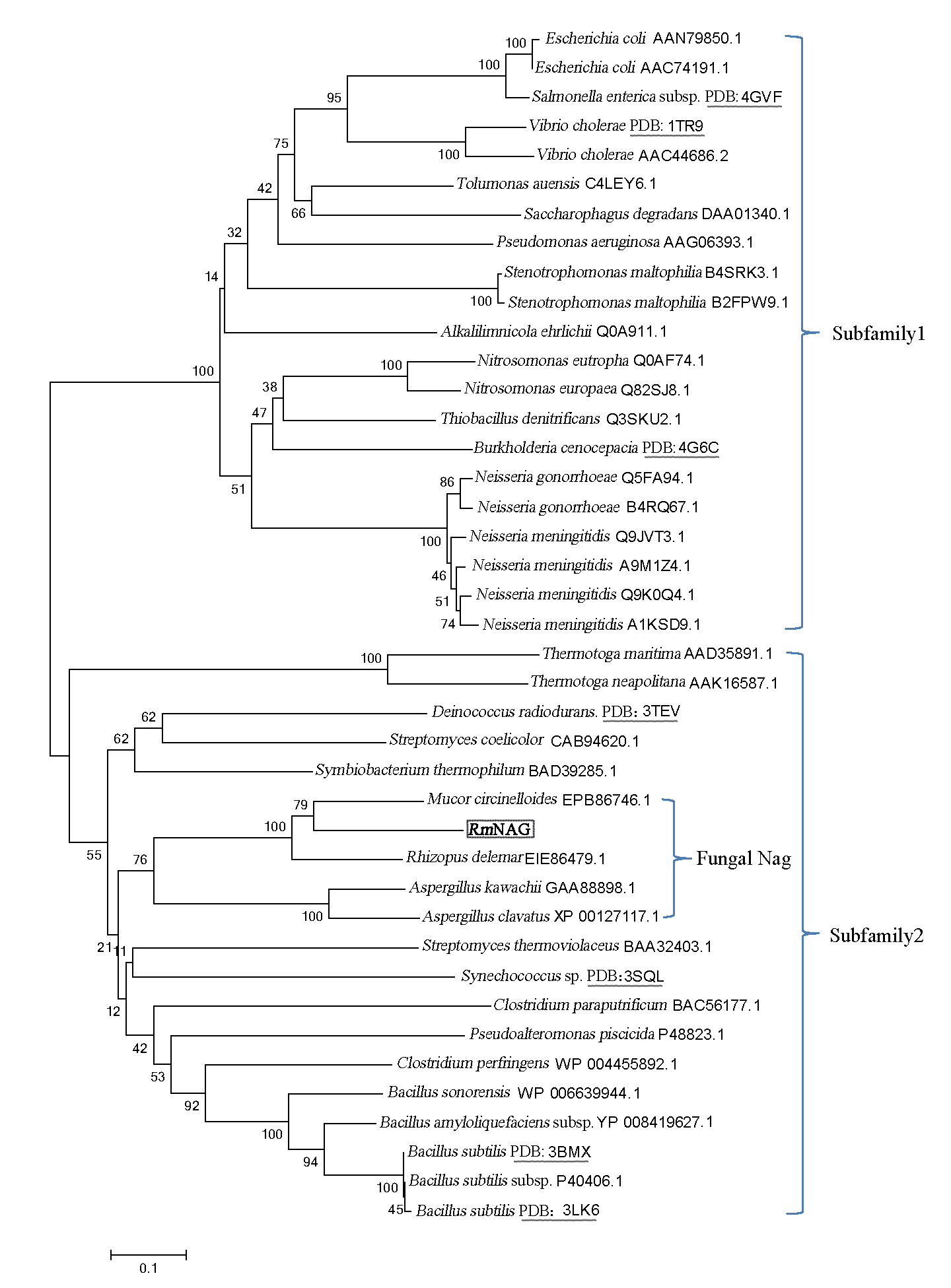


**Supplementary Figure S3**

Phylogenetic tree of GH family 3 β-N-acetylglucosaminidases. The neighbor-joining tree shows phylogenetic relationships between *Rm*Nag and other Genebank entries. The sequence alignment was created with MUSCLE*.* Bootstrap values are expressed as percentages of 1,000 replications. The scale bar indicates a branch length. The phylogenetic tree was constructed using the neighbor-joining method with program MEGA4.
